# ^123^I-Labelled metaiodobenzylguanidine for the evaluation of cardiac sympathetic denervation in early stage amyloidosis

**DOI:** 10.1007/s00259-012-2187-8

**Published:** 2012-07-18

**Authors:** Walter Noordzij, Andor W. J. M. Glaudemans, Ronald W. J. van Rheenen, Bouke P. C. Hazenberg, René A. Tio, Rudi A. J. O. Dierckx, Riemer H. J. A. Slart

**Affiliations:** 1 Department of Nuclear Medicine and Molecular Imaging, University Medical Center Groningen, University of Groningen, PO Box 30.001, 9700 RB Groningen, The Netherlands; 2 Department of Rheumatology and Clinical Immunology, University Medical Center Groningen, University of Groningen, Groningen, The Netherlands; 3 Department of Cardiology, University Medical Center Groningen, University of Groningen, Groningen, The Netherlands

**Keywords:** MIBG, Cardiac, Amyloidosis, Echocardiography

## Abstract

**Purpose:**

Cardiac amyloidosis is a rare disorder, but it may lead to potentially life-threatening restrictive cardiomyopathy. Cardiac manifestations frequently occur in primary amyloidosis (AL) and familial amyloidosis (ATTR), but are uncommon in secondary amyloidosis (AA). Echocardiography is the method of choice for assessing cardiac amyloidosis. Amyloid deposits impair the function of sympathetic nerve endings. Disturbance of myocardial sympathetic innervations may play an important role in the remodelling process. ^123^I-MIBG can detect these innervation changes.

**Methods:**

Patients with biopsy-proven amyloidosis underwent general work-up, echocardiography and ^123^I-MIBG scintigraphy. Left ventricular internal dimensions and wall thickness were measured, and highly refractile cardiac echoes (sparkling) were analysed. Early (15 min) and late (4 h) heart-to-mediastinum ratio (HMR) and wash-out rate were determined after administration of MIBG.

**Results:**

Included in the study were 61 patients (30 women and 31 men; mean age 62 years; 39 AL, 11 AA, 11 ATTR). Echocardiographic parameters were not significantly different between the groups. Sparkling was present in 72 % of ATTR patients, in 54 % of AL patients and in 45 % of AA patients. Mean late HMR in all patients was 2.3 ± 0.75, and the mean wash-out rate was 8.6 ± 14 % (the latter not significantly different between the patient groups). Late HMR was significantly lower in patients with echocardiographic signs of amyloidosis than in patients without (2.0 ± 0.70 versus 2.8 ± 0.58, *p* < 0.001). Wash-out rates were significantly higher in these patients (−3.3 ± 9.9 % vs. 17 ± 10 %, *p* < 0.001). In ATTR patients without echocardiographic signs of amyloidosis, HMR was lower than in patients with the other types (2.0 ± 0.59 vs. 2.9 ± 0.50, *p* = 0.007).

**Conclusion:**

MIBG HMR is lower and wash-out rate is higher in patients with echocardiographic signs of amyloidosis. Also, ^123^I-MIBG scintigraphy can detect cardiac denervation in ATTR patients before signs of amyloidosis are evident on echocardiography.

## Introduction

Amyloidosis comprises a group of diseases characterized by deposition of protein fibrils with a cross-β-pleated sheet molecular structure. The deposition of these amyloid fibrils results in loss of organ function. Even though cardiac amyloidosis seems to be rare, in the majority of patients, amyloidosis can be complicated by cardiac involvement. About 50 % of all amyloidosis patients experience some cardiac manifestations related to the disease [1]. It is frequent in primary amyloidosis (immunoglobulin light chain, or AL type) and familial amyloidosis (transthyretin, or ATTR type, which leads to familial amyloidotic polyneuropathy, FAP), but uncommon in secondary amyloidosis (serum amyloid A protein, or AA type). Up to 90 % of all patients with AL amyloidosis have cardiac manifestations, but only 5 % have clinically isolated cardiac disease. In ATTR amyloidosis, cardiac involvement leads less frequently to systolic dysfunction and heart failure. Furthermore, symptoms are milder and progression is slower, when compared to AL amyloidosis.

Cardiac involvement eventually leads to a type of cardiomyopathy in which ventricular filling is restricted, resulting in symptoms and signs of heart failure. Heart failure occurs in at least 25 % of all patients [1]. The presence of heart failure is associated with a median survival of only 6 months [1]. Amyloidosis is the most common cause of this co-called ‘restrictive cardiomyopathy’. Transthoracic echocardiography plays an important role in the evaluation of cardiac manifestations of amyloidosis. Nowadays it is the modality of choice for the evaluation of amyloid deposition in the heart [2]. The most common findings are left ventricular (LV) wall thickening due to amyloid deposition in the myocardium and highly refractile cardiac echoes (sparkling). This is often associated with right ventricular wall thickening, diffuse valvular infiltration, dilated atria and pericardial effusion [3]. However, with this technique, the diagnosis of cardiac amyloidosis is often established when the disease has already reached a relatively advanced stage, where irreversible functional and structural myocardial changes have occurred (remodelling). Disturbance of myocardial sympathetic innervation may play an important role in this remodelling process, and may even lead to sudden death due to fatal arrhythmia [2]. Amyloid deposits impair the function of myocardial sympathetic nerve endings.

Scintigraphic imaging using ^123^I-MIBG is a validated method to evaluate sympathetic innervation in the heart, and has also been used in patients with amyloidosis [4–6]. ^123^I-MIBG, derived by chemical modification of the false neurotransmitter analogue guanethidine, and therefore an analogue of norepinephrine (NE), enters the sympathetic nerve terminals through a specific “uptake-1 mechanism”. Unlike NE, ^123^I-MIBG is stored in granules in the nerve terminals and is not catabolized. In various case studies and trials, defects on ^123^I-MIBG scans have been found to represent impaired cardiac sympathetic nerve endings due to amyloid deposition [4–9]. So, ^123^I-MIBG indirectly visualizes the effect of amyloid deposition in the myocardium [10]. This technique might be able to detect early cardiac denervation before actual heart failure occurs, and is also thought to visualize impaired sympathetic innervation before abnormalities appear on echocardiography.

The purpose of this study was to determine if ^123^I-MIBG scintigraphy is able to identify cardiac sympathetic denervation in patients with different types of early stage amyloidosis.

## Materials and methods

### Patients

A total of 63 consecutive patients (31 women, 32 men) with systemic amyloidosis underwent ^123^I-MIBG scintigraphy between June 2007 and August 2011. Diagnosis of amyloidosis was based on detection of amyloid in a biopsy site typically involved in systemic amyloidosis, such as abdominal fat tissue, kidney or nerve. One patient was excluded because of insufficient histological proof of amyloid in the biopsy. History and physical examination were used to determine the presence of polyneuropathy. All patients underwent the usual tests including 12-lead electrocardiography (ECG), dynamic electrocardiography (Holter investigation), radionuclide multiple gated acquisition (MUGA) for LV ejection fraction (LVEF), ^123^I-labelled serum amyloid P scintigraphy to evaluate total body amyloid deposition, autonomic function testing using bedside manoeuvres, heart rate variability analysis and echocardiographic examination before ^123^I-MIBG scintigraphy.

For ^123^I-MIBG imaging, nine age-matched consecutive normal volunteers were scanned to form a healthy control database. All subjects were in good health and were not taking medication, especially tricyclic antidepressants or other sympathicomimetics that can interfere with ^123^I-MIBG uptake.

### ^123^I-MIBG scintigraphy

Scintigraphy was performed after blockade of thyroid uptake of free ^123^I by iodine potassium iodide (Lugol’s solution). After a 15-min rest period, subjects were injected with 185 MBq ^123^I-MIBG (AdreView; GE Healthcare Medical Diagnostics, Eindhoven, The Netherlands) through an intravenous catheter. At 15 min (early image) and 4 h (delayed image) after tracer administration, 10-min static anterior view images of the chest were acquired using a Symbia S gamma camera (Siemens Medical Systems, Knoxville, TN) with a medium energy low-penetration parallel-hole collimator, as recommended in the proposal for standardization of ^123^I-MIBG scintigraphy for cardiac sympathetic innervation imaging [11]. A 15 % energy window centred on 159 keV, a 256 × 256 matrix size and a 1.45 zoom factor was used.

### Multiple gated equilibrium radionuclide angiography

MUGAs were standardized and performed in the left anterior oblique projection after in vivo labelling of red blood cells with 750 MBq of ^99m^Tc-pertechnetate to determine LVEF. Images were collected in a 64 × 64 matrix in 20 frames/cycle during a 10-min acquisition. The Symbia S gamma camera with a low-energy all-purpose collimator was used. The camera head was positioned in the best septal left anterior oblique projection, typically with a caudal tilt of 5–10°. R-wave triggering was performed in a 20 % beat acceptance window with two-thirds forward and one-third backward framing per cardiac cycle, for 20 frames per R-R interval for a total of 6 min. Data were acquired using 64 × 64 matrices in a 15 % energy window centred on the 140 keV photopeak. Processing was performed on dedicated computers (Syngo MI; Siemens Medical Systems). For each of the 20 frames a region of interest (ROI) was automatically drawn around the LV using a validated, fully automated, operator-independent, contour detection algorithm. Frames were automatically corrected for background activity. Background activity ROIs were generated automatically. All LVEF values were generated without decimals and are highly reproducible [12].

### ^123^I-MIBG scintigraphy image analysis

For planar images, LV activity was measured over the raw static image using a ROI along the contour of the LV. A second ROI was placed over the upper mediastinal area. The heart-to-mediastinum activity ratio (HMR) was measured three times, and the measurements averaged. The cardiac ^123^I-MIBG wash-out rate was defined as the percentage change in activity ratio from the early to the late images within the LV ROI as follows: [(HMR_early_ − HMR_late_)/HMR_early_] × 100 %, data being corrected for the physical decay of ^123^I. Data were also compared with our local normal database.

### Heart rate variability analysis

Heart rate variability based on the 24-h Holter electrocardiogram was analysed during hospitalization. The following nonspectral indices (time domain) were computed and expressed in milliseconds: the average NN interval of normal cycles; its standard deviation (SDNN), which reflects all the cyclic components responsible for variability; and the SDNN index, which is the mean of all 5-min standard deviations of NN intervals during a 24-h period.

### Echocardiographic examination

The following variables were measured according to standard recommendations in the M-mode echocardiographic examination: LV internal end-diastolic and end-systolic dimensions, interventricular wall thickness and LV posterior wall thickness at end-diastole. In the two-dimensional echocardiographic examination, highly refractile cardiac echoes, the so-called granular sparkling appearance, were sought. Using Doppler recordings of transmitral flow velocity, the ratio of peak flow velocity of LV rapid diastolic filling (peak E) to peak flow velocity during atrial contraction (peak A) was measured, and a ratio lower than 1 was considered abnormal. A visually estimated LVEF of >55 % was considered normal, between 55 % and 40 % mildly disturbed, between 30 % and 40 % moderately disturbed, and <30 % severely impaired systolic function.

### Statistical analysis

The results are expressed as means ± standard deviation, or median (range) if the data were not normally distributed. The differences between patient categories were evaluated using unpaired Student’s *t*-tests. *P* values <0.05 were considered significant.

## Results

### Patient characteristics

The baseline characteristics of the patients (31 women and 32 men) are summarized in Table 1. Their mean age was 62 ± 8.8 years. The mean age of the 9 healthy controls (6 women and 3 men) was not significantly different (52 ± 17 years). Of all 63 patients, 39 were diagnosed with AL amyloidosis, 11 with AA type and 11 with ATTR type. In one patient, the type of amyloidosis remained unclear, despite extensive testing. One patient was excluded because the presence of amyloidosis could not be confirmed histologically. There was no difference in medication use between the different patient groups. Polyneuropathy was present in 19 patients (10 AL, 1 AA and 8 ATTR). Clinical evidence of autonomic dysfunction was present in 25 patients (18 AL, 2 AA and 5 ATTR). The clinical signs of heart failure are also presented in Table 1. No significant difference between the different patients groups was found regarding these parameters. ECG abnormalities at baseline included left axis deviation, signs of LV hypertrophy, conduction delay (QRS >110 ms), and microvoltage in standard leads. Atrioventricular block recorded on Holter registration was infrequent (ten patients): five first-degree and 1 s-degree in AL patients and five in ATTR patients. The QRS complex was significantly longer in ATTR patients: 104 ms versus 88 ms in AA patients as well as AL patients (*p* < 0.005), but still within the normal range (<110 ms). Ventricular tachycardia (VT) occurred significantly more often in ATTR patients (median 1, range 0–13). However, the number of VTs over five beats was the same in AL and ATTR patients (5). Median LVEF on MUGA was 60 % (range 29–70 %), and did not differ between the groups. The presence of wall motion abnormalities on MUGA was not different between the groups.

**Table 1:** Patient characteristics at baseline

Characteristic	AL patients	AA patients	ATTR patients
Gender (*n*)			
Male	21	3	7
Female	18	8	4
Age (years, mean ± SD)	62 ± 8.8	64 ± 13	56 ± 13
Heart biopsy (*n*)	0	0	2
Polyneuropathy (*n*)	10	1	8
Autonomic neuropathy (*n*)	18	2	5
Signs of heart failure (*n*)			
Cor-thorax ratio >50 (*n*)	6	1	4
ECG abnormalities (*n*)	12	3	7
NYHA class for heart failure (*n*)			
I	22	8	8
II	17	3	3
III	0	0	0
IV	0	0	0
Hypertension(*n*)	27	7	5
Oedema (*n*)	15	3	3
Known coronary artery disease (*n*)	4	0	0
Medication at baseline (*n*)			
Beta-blocker	7	3	3
ACE inhibitor	8	3	1
Antiepileptic agents	4	1	1
Selective serotonin reuptake inhibitors	2	1	1
Angiotensin-2 antagonists	3	2	2
Alpha-1 blocker	0	1	0
Laboratory values at presentation, median (range)			
NT-proBNP (ng/l)	926 (38–59,544)	792 (29–38,308)	777 (80–2,596)
Troponin T (μg/l)	<0.05 (<0.05-1.32)	0.01 (<0.05–0.08)	<0.05 (<0.05–4.0)
MUGA parameters, median (range)			
LVEF (%)	60 (32–70)	65 (51–67)	56 (29–65)
Wall motion abnormalities LV (*n*)			
None	34	8	5
Diffuse	3	1	1
Regional	2	0	3
RVEF (*n*)			
Normal	35	9	8
Mildly disturbed	3	0	0
Moderately disturbed	0	0	1

### ^123^I-MIBG scintigraphy

**Fig. 1:**
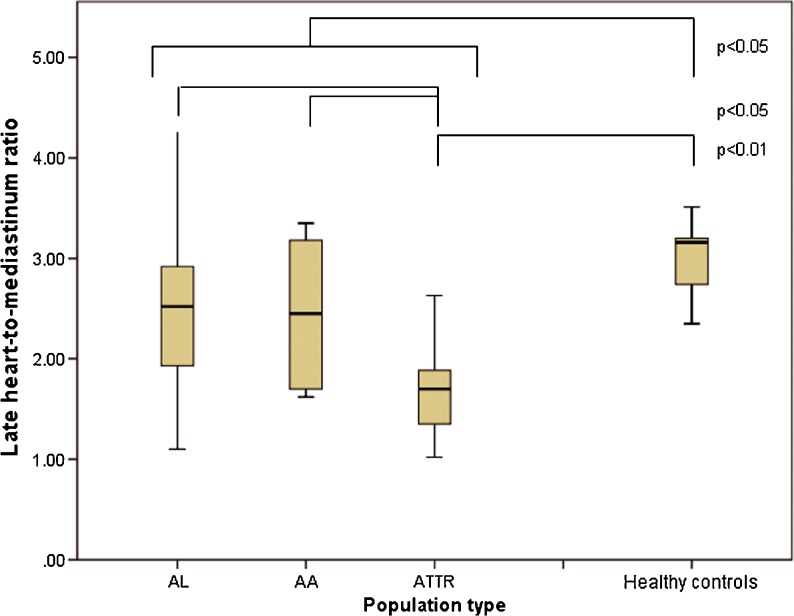
Late HMR for all patient groups and the healthy controls

**Fig. 2:**
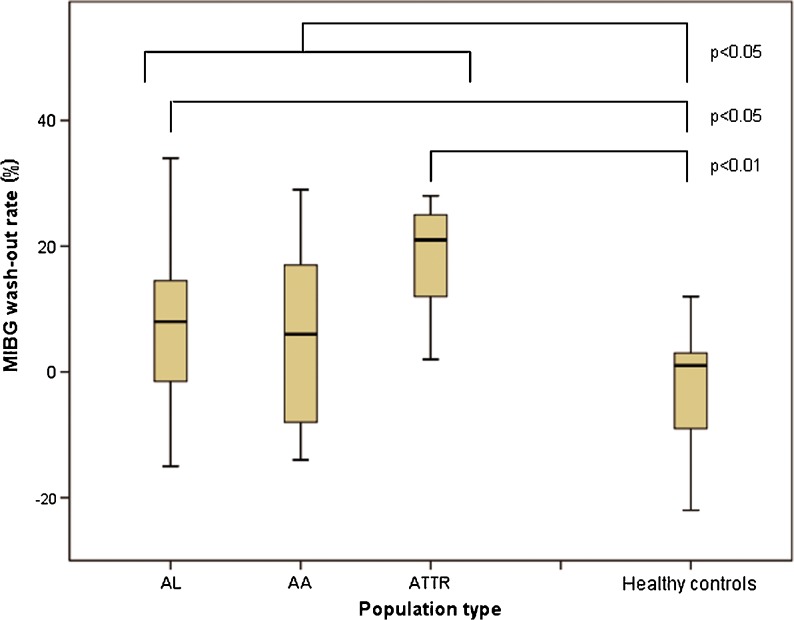
^123^I-MIBG wash-out rates for all patient groups and the healthy controls

^123^I-MIBG scans were performed within 186 days (0-412 days) after the date that the presence of amyloidosis was confirmed by histology. Table 2 summarizes the results of the ^123^I-MIBG scans in the different patient groups and the healthy controls. In all patients the HMR was significantly lower than in healthy controls (mean HMR 2.3 ± 0.75 vs. 2.9 ± 0.58, *p* < 0.005; Fig. 1) Furthermore, the wash-out rate in all patients was significantly higher than in healthy controls (mean wash-out rate 8.6 ± 14 % vs. −2.1 ± 10 %, *p* < 0.05; Fig. 2). Among all patients, the mean HMR did not differ significantly between those with and without polyneuropathy or autonomic neuropathy. The wash-out rates in patients with polyneuropathy were significantly higher (14 ± 10.7 vs. 6.6 ± 15, *p* < 0.05)), but not in patients with autonomic neuropathy. The HMR was significantly lower in ATTR patients (1.7 ± 0.52) than in the other groups (2.5 ± 0.75 in AL patients and 2.4 ± 0.75 in AA patients, *p* < 0.05). In ATTR patients the HMR was significantly lower than in healthy controls (*p* < 0.001), but not different from that in AL or AA patients.

**Table 2:** ^123^I-MIBG findings

	Healthy controls	AL patients	AA patients	ATTR patients
Late HMR, mean ± SD or median (range)	2.9 ± 0.58	2.5 ± 0.75	2.4 ± 0.75	1.7 (1.0–2.6)
Wash-out rate (%, mean ± SD)	−2.1 ± 10	7.0 ± 14	5.9 ± 14	18 ± 8.3
Wash-out rate cut-off at 0 (%, mean ± SD)	2.6 ± 3.8	9.6 ± 11	8.9 ± 11	18 ± 8.5
Wash-out rate >20 % (*n*)	0	7	2	6

The wash-out rate in healthy controls was significantly lower than in the patients overall. It was also significantly lower than in the ATTR patients (*p* < 0.001) and in the AL patients (*p* < 0.005). There was no difference in wash-out rate between the three patients groups. Using a wash-out rate ≥0 %, the rate in ATTR patients was significantly higher than in patients with other types of amyloidosis (*p* < 0.05). Using this cut-off, the wash-out rate in healthy controls was significantly lower than in the patients overall (2.6 ± 3.8 % vs. 11 ± 11 %, *p* < 0.001). A wash-out rate higher than 20 % occurred significantly more often in AL patients and ATTR patients (*p* < 0.05) than in AA patients.

Patients with signs of heart failure, specifically a cor-thorax ratio >50, showed significantly lower HMR (1.78 ± 0.54 vs. 2.46 ± 0.74, *p* = 0.003) and higher wash-out rates (23 ± 6.8 % vs. 5.8 ±13 %, *p* < 0.001) than those without an enlarged heart on conventional chest radiographs. Also patients with ECG abnormalities at baseline showed lower HMR (1.91 ± 0.60 vs. 2.55 ± 0.74, *p* = 0.001) and higher wash-out rates (15 ± 12 % vs. 5.1 ± 14 %, *p* = 0.004) than those with a normal ECG.

### Cardiac denervation–heart rate variability relationship

There was no difference in SDNN among the three patient groups, nor in the SDNN index.

### Echocardiography

Echocardiography and ^123^I-MIBG scans were performed within 81 ± 141 days consecutively. Table 3 summarizes the echocardiographic examination results. Left and right ventricular wall thickness was overall normal in the different groups, except for a slightly thicker posterior LV wall in the ATTR patients. LV diastolic function was mildly disturbed more frequently in AL patients. However, there were no significant differences in the frequency of diastolic dysfunction among the different groups. The HMR and wash-out rate were also not different between patients with and without diastolic dysfunction on echocardiography. The visually estimated LVEF was mostly normal in all three patient groups. LVEF measured by MUGA corresponded well with visually estimated LVEF. The combination of LVEF <40 % and the presence of parameters of amyloidosis on echocardiography occurred significantly more often in the ATTR patients (36 %).

**Table 3:** Echocardiographic results

	AL patients	AA patients	ATTR patients
Wall thickness (mm), median (range)			
Septal wall	12 (7–23)	12 (8–18)	13 (7–20)
Posterior wall	11 (8–19)	11 (9–15)	16 (8–20)
Right ventricle wall	5 (3–10)	6 (4–8)	6 (4–9)
Diastolic function (*n*)			
Inaccessible	3	2	0
Normal	5	4	2
Mildly disturbed	22	5	3
Moderately disturbed	5	1	3
Severely disturbed	4	1	1
Amyloid parameters on echocardiogram^a^ (*n*)	21	5	8
LVEF on echocardiogram (*n*)			
Normal	25	8	6
Mildly disturbed	11	3	3
Moderately disturbed	3	0	1
Severely disturbed	0	0	1
Cardiomyopathy^b^ (*n*)	3	0	4

^a^Sparkling and LV wall thickness >11 mm.

^b^Amyloid + LVEF <40 %.

Characteristic sparkling on echocardiography appeared in 73 % of the ATTR patients, and in 54 % and 45 % in the AL and AA patients, respectively. Of the AL patients, three had the combination of LVEF <40 % and echocardiographic parameters of amyloidosis. This combination occurred significantly more often (*p* < 0.05) in the ATTR patients (4 out of 11).

**Fig. 3:**
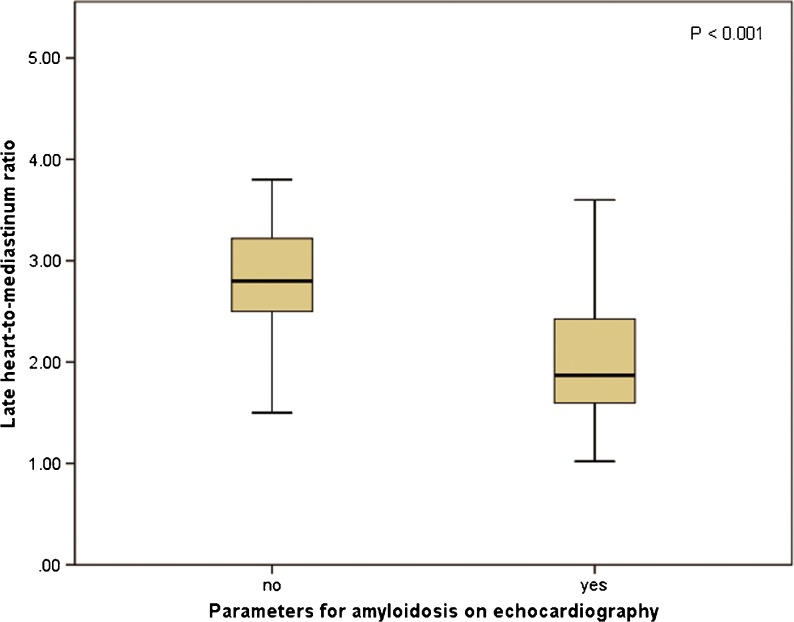
Late HMR in patients with and without echocardiographic signs of amyloidosis

**Fig. 4:**
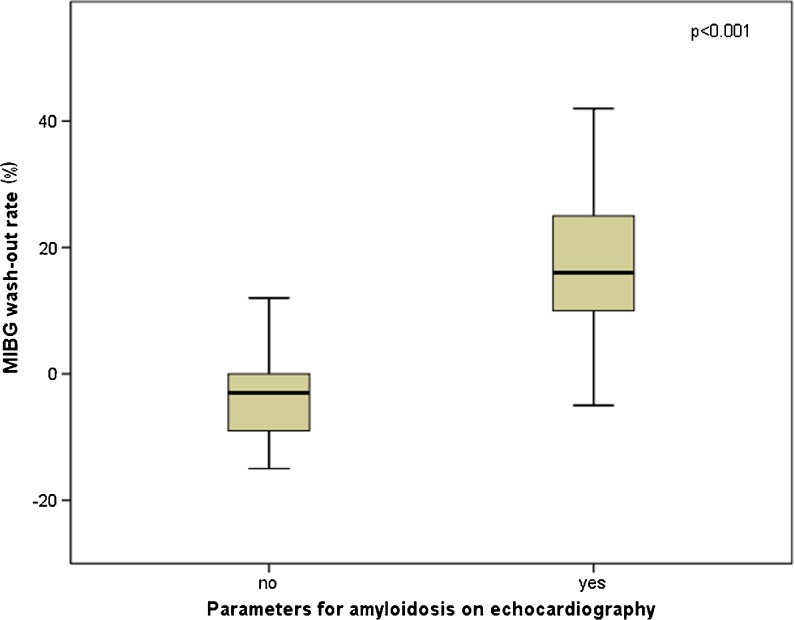
^123^I-MIBG wash-out rates in patients with and without echocardiographic parameters for amyloidosis

Late HMR was significantly different between patients with and without echocardiographic parameters for amyloidosis, defined as sparkling and LV wall thickness >11 mm (2.0 ± 0.70 vs. 2.8 ± 0.58, *p* < 0.001; Fig. 3). Also, the wash-out rate was significantly higher in these patients (−3.3 ± 9.9 % vs. 17 ± 10 %, *p* < 0.001; Fig. 4). There was no difference in HMR or wash-out rate among the three patient groups in patients with echocardiographic parameters of amyloidosis. However, in ATTR patients without echocardiographic parameters of amyloidosis, HMR was lower than in patients with the other types (2.0 ± 0.59 vs. 2.9 ± 0.50, *p* = 0.007). Furthermore, wash-out rates were higher in these patients (11 ± 1.4 % vs. −4.6 ± 9.3 %, *p* = 0.03). There was also no difference in polyneuropathy or autonomic neuropathy in patients with echocardiographic signs of amyloidosis.

## Discussion

Our results indicate that ^123^I-MIBG scintigraphy can be used to determine cardiac sympathetic denervation in patients with early stage amyloidosis. Furthermore, in ATTR patients cardiac denervation can be found even before echocardiographic parameters are present. To our knowledge, this is the first study to find a significant difference between subgroups of amyloidosis patients in echocardiographic findings of cardiac amyloidosis, and HMR and wash-out rates determined by ^123^I-MIBG scintigraphy prior to an intervention. Sympathetic denervation, determined by diminished late ^123^I-MIBG uptake in HMR and elevated wash-out rate, was prominent in patients with echocardiographic parameters for amyloidosis. Furthermore, HMR was lower and wash-out rates higher in patients with amyloidosis than in healthy controls.

In previous studies involving ^123^I-MIBG in patients with amyloidosis, echocardiography was also performed. The slight increase in wall thickness in ATTR patients and in those with FAP is in accordance with the findings of previous studies [6–8]. In AL patients thickened ventricular walls have been found, but only in a minority [5]. However, in patients with FAP who had undergone liver transplantation, an inverse correlation has been found between septal and posterior wall thickness determined by echocardiography and cardiac ^123^I-MIBG uptake after liver transplantation [8]. These authors did not find a correlation before liver transplantation or between echocardiographic changes and changes in ^123^I-MIBG uptake after the intervention.

About 50 % of all amyloidosis patients experience cardiac manifestations related to the disease, most frequently in those with AL or ATTR. Symptoms and consequences of cardiac amyloid deposition in AL amyloidosis are often more frequent and severe than in FAP (causing more fatal dysfunction). Myocardial defects in ^123^I-MIBG activity correlate with impaired cardiac sympathetic function due to amyloid deposition. This can be identified early in the disease. Several studies have shown the value of HMR and wash-out rate as indicators of sympathetic innervation abnormalities in cardiac amyloidosis. The use of ^123^I-MIBG (the best reported imaging modality for cardiac sympathetic denervation) is highly reproducible and is an easily accessible method, making it not readily substituted by other modalities. Furthermore, lower HMR and higher wash-out rates correspond to severity of the disease.

The use of ^123^I-MIBG has been studied most intensively in patients with FAP. The ^123^I-MIBG results in this study were generally less aberrant than those in previous studies [4–6, 8]. Not only did the healthy controls have higher HMR than in previous studies, but our amyloidosis patients also showed higher ^123^I-MIBG uptake and less wash-out. In the study of patients with FAP who underwent liver transplantation, the mean HMR was 1.45 ± 0.29 before and 1.46 ± 0.28 after liver transplantation. Also the wash-out rate in these patients (28.5 ± 9.1 % and 24.6 ± 9.6 %, respectively) was higher than in our group. The most important explanation of the large difference lies in the collimator type used. The previous studies all used low-energy collimators, whereas in this study a medium-energy collimator was used. The choice of collimator type influences the HMR, with higher HMR on ^123^I-MIBG scintigraphy with a medium-energy collimator than with a low-energy collimator for scans performed in the same setting [13]. Another explanation may be the use of different ^123^I-MIBG specific activities in Japan, the US and The Netherlands. In this study the specific activity of ^123^I-MIBG was 74 MBq/ml (Adreviwew, GE Healthcare). Unfortunately, the specific activity of ^123^I-MIBG used in previous studies is not available.

The use of ^123^I-MIBG in AL type amyloidosis has been less intensively studied. Only one major study has been performed in which the presence of impaired myocardial sympathetic innervation was found to be related to clinical autonomic abnormalities and congestive heart failure in AL amyloidosis [9]. The mean late HMR in our group of AL patients was higher than the mean value in the control group in the previous study in patients before liver transplantation [6]. The wash-out rate in the control group in the previous study was generally over 20 %, whereas in our patients the median wash-out rate was 8 %. This may indicate either that different ethnic groups have different ^123^I-MIBG kinetics or that patients with early stage amyloidosis, as in our patients, have a less-intense wash-out of ^123^I-MIBG than patients with later stage amyloidosis.

In our patients, no relationship was found between ^123^I-MIBG uptake and autonomic dysfunction on bedside manoeuvres, or peripheral neuropathy. In other groups with both FAP and AL type amyloidosis, patients with either autonomic or peripheral neuropathy had lower late HMR and higher wash-out rates. This may also be due to the early stage of the amyloidosis in our patients. This may imply a differential autonomic neuropathy, which means that cardiac autonomic dysfunction cannot be ruled out in those with normal peripheral autonomic function. Despite the fact that ten of our AL patients suffered from polyneuropathy and 18 suffered from autonomic neuropathy, we were not able to identify a correlation between the presence of neuropathy and low HMR or high wash-out rate. This may have been due to the lower test characteristics of the autonomic function tests. Furthermore, no significant difference in HMR or wash-out rate between the AL and AA patients was found. This can be explained partly by the fact that fewer AA patients show cardiac involvement. Another explanation may be the fact that ATTR patients suffer from more severe cardiac manifestations in the early stage of the disease. This is in contrast to the general opinion that amyloidotic cardiomyopathy in ATTR patients is less aggressive than in AL patients [14]. However, the prognosis in ATTR patients depends on the type of mutation, age of onset and severity of cardiac manifestations [15]. Since the age of the patients in the different groups was not significantly different, the explanation may be found in the severity of cardiac disease and the type of mutation.

In our study, SPECT imaging was not performed routinely. SPECT imaging has been reported to have advantages for evaluating abnormalities in regional distribution in the myocardium [4–9]. Usually, the reconstructed data are displayed in three planes (short axis, horizontal long axis and vertical long axis), which is similar to that used in myocardial perfusion SPECT. Various patterns of distribution of myocardial ^123^I-MIBG accumulation have been reported, but the quality of ^123^I-MIBG SPECT images is moderate due to the properties of the tracer. Furthermore, no myocardial perfusion scans were performed. Comparing perfusion imaging to ^123^I-MIBG distribution gives extra information about the presence or absence of mismatch patterns. Myocardial ischaemia or infarction disrupts sympathetic transmission, which may lead to denervation of a region larger than that affected by ischaemia only. Furthermore, sympathetic nervous tissue is more sensitive to ischaemia than myocytes. The presence of innervation/perfusion imaging mismatches correlates with electrophysiological abnormalities and increasing inducibility of potential lethal arrhythmias [16, 17].

### PET in cardiac denervation

Various PET analogues of NE are also under investigation [18]. These are even more similar to NE than ^123^I-MIBG and show advantages for imaging. ^11^C-meta-hydroxy-ephedrine (^11^C-mHED) is the most commonly used PET tracer. It has a higher sensitivity for the uptake-1 mechanism than ^123^I-MIBG, and is not linked to the uptake-2 mechanism, which might allow better differentiation between innervated and denervated myocardium. In a group of 21 patients with LV dysfunction who underwent both ^123^I-MIBG and ^11^C-mHED imaging, the correlation between MIBG wash-out rate and mHED wash-out rate was poor (*r* = 0.57), but the defect sizes on both early (*r* = 0.94) and late images (*r* = 0.88) with these two modalities were more closely associated. Therefore, ^11^C-mHED seems to have advantages over ^123^I-MIBG in the detection of regional abnormalities [19]. Other ^11^C-labelled tracers, including ^11^C-phenethylguanidine, are currently under investigation. Promising results from a study in rats show that ^11^C-phenethylguanidine and its analogues are transported more slowly that MIBG and mHED, and therefore might provide more accurate measurement of cardiac nerve density [20].

To the best of our knowledge no PET tracers have been used to visualize cardiac denervation in patients with cardiac manifestations of amyloidosis.

### Clinical implications

The results and measurements in echocardiography depend on the level of experience of the user and this may lead to interobserver variability. Experienced users may be able to detect cardiac amyloidosis accurately and therefore at an early stage of the disease. The fact that in patients with echocardiographic parameters for amyloidosis, late ^123^I-MIBG uptake was lower and wash-out rate was higher shows that the two investigations may complement each other. Amyloidosis with cardiac involvement is a difficult diagnosis and one imaging technique may confirm the diagnosis or add value to the other. Both types of investigations are well established in the work-up in patients with amyloidosis and should be performed routinely.

Another point of interest is the role of ^123^I-MIBG in predicting sudden cardiac death in patients with heart failure and even the predictive value of appropriate implantable cardioverter-defibrillator (ICD) therapy. Several studies indicate that lower HMR is associated with an increased likelihood of an ICD discharge [21, 22]. ^123^I-MIBG scanning may also be able to play a role in decision making concerning ICD therapy in patients with heart failure due to cardiac amyloidosis.

### Conclusion

Patients with biopsy-proven amyloidosis and echocardiographic findings of cardiac manifestations showed significantly lower HMR and higher wash-out rate than patients without these findings. Furthermore, ^123^I-MIBG scintigraphy was able to detect cardiac sympathetic denervation in ATTR patients before echocardiographic evidence was apparent. ^123^I-MIBG scanning may successfully detect cardiac sympathetic denervation in all patients with early stage amyloidosis. Finally, ^123^I-MIBG and echocardiography may complement each other in the diagnosis of cardiac manifestations of amyloidosis, and should be performed consecutively in a routine manner.
